# Current Best Practices and Emerging Approaches in the Management of Acute Spinal Trauma

**DOI:** 10.1155/2019/8634260

**Published:** 2019-01-01

**Authors:** Kivanc Atesok, Nobuhiro Tanaka, Yohan Robinson, Jason Pittman, Steven Theiss

**Affiliations:** ^1^Harvard University, Beth Israel Deaconess Medical Center, Department of Neurosurgery, Boston, MA, USA; ^2^Hiroshima General Hospital, Department of Orthopaedic Surgery, Hiroshima, Japan; ^3^Armed Forces Centre for Defense Medicine, Department of Research and Development, Gothenburg, Sweden; ^4^University of Alabama at Birmingham, Department of Orthopaedic Surgery, Birmingham, AL, USA

The past few decades have seen tremendous developments in surgeons' approaches to patients with acute traumatic injuries involving the spinal column and the spinal cord. As a result of advances in the scientific knowledge of the biomechanical and neurological bases of spinal trauma, along with ever-improved imaging modalities and operating techniques, nonsurgical and surgical management approaches to spine trauma patients have evolved. Due to its unique, diverse, and also challenging aspects, spinal trauma must be handled by orthopaedic surgeons and neurosurgeons with subspecialty training or particular expertise in spine surgery.

This special issue presents articles focusing on current concepts in the management of acute traumatic spinal injuries. These papers cover a broad range of spine trauma topics. A brief synopsis of the papers is as follows.

In a remarkable study of 6,370 patients with C2 fractures from the Swedish National Registry, A.-L. Robinson et al. underline the dynamic changes in the epidemiology of axis fractures that may result from changing population demographics, such as increasing age and level of activity. K. Atesok et al. critically evaluate the available evidence regarding posttraumatic spinal-cord injury without radiographic abnormality (SCIWORA). These expert authors have provided the most up-to-date algorithm for the management of patients with SCIWORA.

Whiplash is the most common injury associated with motor-vehicle accidents, affecting up to 83% of patients involved in collisions and imposing an overall economic burden of $3.9 billion annually in the US [1]. In light of the impact of whiplash on human health and healthcare systems, N. Tanaka et al. performed a literature review that points out the need for more comprehensive guidelines for addressing the diversity of the syndrome.

One of the pressing issues in the surgical treatment of fresh osteoporotic fractures with vertebroplasty or balloon kyphoplasty is the challenge of using cement that does not osteointegrate and is also associated with complications such as leakage, embolus, and a high setting temperature. P. Korovessis et al. compare the use of polymethacrylate with that of strontium hydroxyapatite (Sr-HA) in patients with single fresh AO-type A2 or A3 thoracolumbar vertebral body fractures who underwent vertebroplasty with PEEK plus short-segment percutaneous pedicle-screw fixation. The authors observed resorption and replacement of Sr-HA with vertebral bone at 12 months after surgery in all the patients treated with Sr-HA.

Traumatic lumbosacral dislocation is a severe, high-energy injury that usually requires surgical treatment. A. S. Moon et al. contribute a literature review summarizing lumbosacral dislocation with regard to the relevant prognosis, management, anatomy, classification schemes, clinical evaluation, and biomechanics of injury. In another interesting research paper on the same topic, Pearson and colleagues compare the outcomes of percutaneous fixation using indirect reduction techniques with open reduction and internal fixation in patients with spinopelvic dissociation. Their results show no significant differences between the two techniques in terms of postoperative spinopelvic radiographic parameters.

Denis zone III sacral fractures involve the spinal canal and are associated with the highest prevalence and severity of neurological injury [2]. Surgical treatment of this fracture type with posterior open-plate fixation and other spinal instrumentation may cause soft-tissue damage and lead to wound complications. H. Irifune et al. join this special issue with a research paper reporting the clinical results of closed reduction in a hyperextended supine position with percutaneous transsacral-transiliac and iliosacral screw-fixation methods in patients with Denis zone III sacral fractures.

Finally, every spine surgeon practicing at a major level-1 trauma center needs to be knowledgeable in the management of gunshot wounds to the spine. Despite the severity and increasing frequency of spinal gunshot injuries, there is little agreement on the universal classification and management of these injuries. Realizing this, the editors of the special issue are pleased to present a fascinating article from J. R. Staggers et al. that critically assesses the utility of trauma-classification systems in such injuries. In their clinical research study, the authors investigated the validity of trauma-classification systems—including the Thoracolumbar Injury Classification and Severity Score (TLICS), Subaxial Cervical Spine Injury Classification and Severity Score (SLIC), and Denis's three-column model—when applied to spinal penetrating trauma from gunshots while secondarily evaluating the stability of these injuries.
